# Supplementary material for: Identification of a Major QTL-Controlling Resistance to the Subtropical Race 4 of Fusarium oxysporum f. sp. cubense in Musa acuminata ssp. malaccensis
Source: Pathogens. 2023 Feb 9;12(2):289. doi: 10.3390/pathogens12020289 (PMC9964652; doi:10.3390/pathogens12020289)
Supplement: Supplementary file 1 [file pathogens-12-00289-s001.zip › pathogens-2123749-supplementary.pdf]

Identification of a major QTL controlling resistance to the Subtropical Race 4 of *Fusarium oxysporum* f. sp. *cubense* in *Musa acuminata* ssp. *malaccensis*.

Supplementary Data

Figure S1.

Table S1.

Table S2.

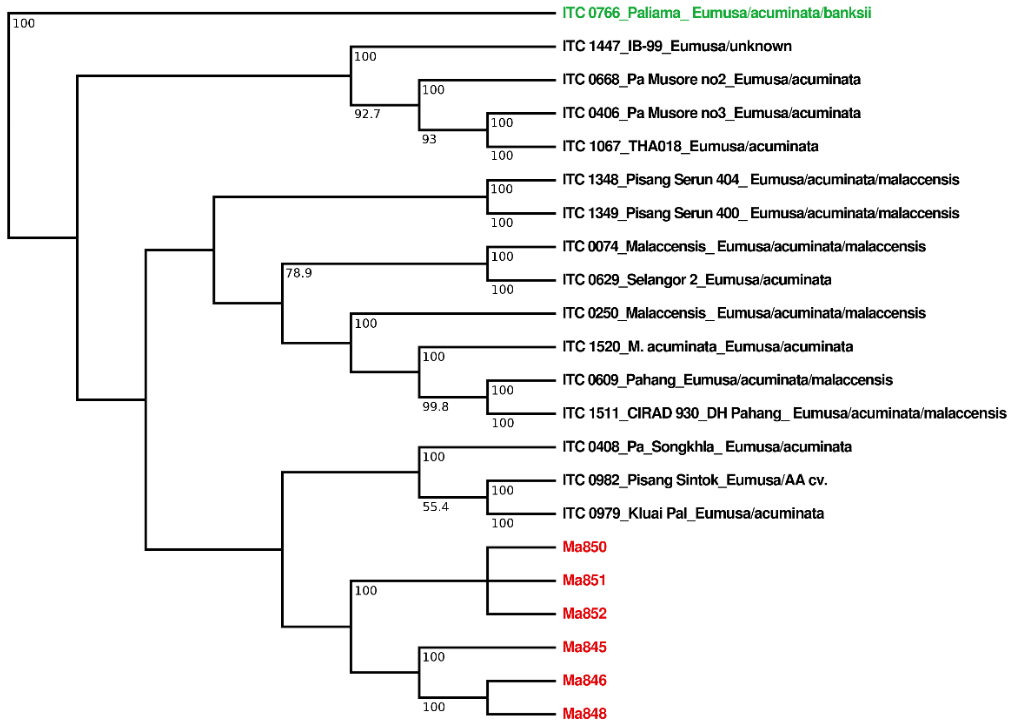

Figure S1. UPGMA analysis of 'Ma845', 'Ma846', 'Ma848', 'Ma850', 'Ma851', 'Ma852' using SSR genotyping and *M. acuminata*. ssp. *malaccensis* accessions from the *Musa* core collection. Bootstrap support values higher than 50% are marked below the corresponding branches. The accessions used in this study are highlighted in red. The *M. a.* ssp. *banksii* used as an outgroup to anchor the whole group is highlighted in green.

Table S1. Annotated effects in Sequence Ontology (SO) terms associated with the variants detected in the 'SnpEff' analysis. The SO numbers are listed next to the terms. The up- and down- stream regions define a 5 Kb sequence extending from the respective 3′ and 5′ UTRs. The splice_region_variant corresponds to a region within 1-3 bp of the exon and 3-8 bp of the intron. The transcript_variant is associated with non-coding transcript variants. The gene_variant is associated with gene fusion variants. Count and percent show the total number of effects and a percentage associated with these terms, respectively.

Effect Seq. Ontology (SO)	Count	Percent
intergenic_region (0000605)	2,083,710	29.37
upstream_gene_variant (0001631)	1,805,574	25.45
downstream_gene_variant (0001632)	1,797,363	25.33
intron_variant (0001627)	1,004,165	14.15
exon_variant (0001791)	263,738	3.72
3_prime_UTR_variant (0001624)	65,819	0.93
5_prime_UTR_variant (0001623)	53,746	0.76
splice_region_variant (0001630)	20,030	0.28
splice_donor_variant (0001575)	642	0.01
splice_acceptor_variant (0001574)	506	0.01
transcript_variant (0001576)	407	0.01
gene_variant (0001564)	10	0

Table S2. A list of putatively defined candidate genes with potential roles in *Foc* STR4 resistance. The genes are retrieved from the DH-Pahang v4.3 assembly. RLK: receptor-like Kinase; RLP: receptor-like protein; NBS: nucleotide binding site; NBS-LRR: nucleotide binding site and leucine-rich repeat; STK: serine/threonine kinase; ERL2: ERECTA-like 2; GSO1: GASSHO1; CEPR2: C-terminally encoded peptide receptor 2; TMK1: transmembrane kinase 1; EIX2: ethylene-inducing xylanase 2; CC: coiled-coil; NB-ARC: nucleotide-binding adaptor shared by APAF-1, R proteins and CED-4; RPP13: recognition of *Peronospora parasitica* (a.k.a *Hyaloperonospora arabidopsidis*) 13; RGA: resistance gene analog; BTB/POZ: broad complex, tramtrack and bric-à-brac/poxirus and zinc finger; AC: adenylyl cyclase; ANK: ankyrin repeats; NPR1: Nonexpressor of pathogenesis-related genes 1. An asterisk (*) next to the gene name denotes the presence of exon SNP variants detected from 'ANNOVAR'. The ^ symbol denotes gene accessions associated with the enriched GO:0043531 (ADP binding) in the GO category of Molecular Function.

Gene Name	Description	Category
Macma4_03_g25720.1	Calcium/calmodulin-regulated receptor-like kinase 1	RLK
Macma4_03_g29370.1	Cysteine-rich receptor-like protein kinase 2	RLK
Macma4_03_g31620.1	Cysteine-rich receptor-like protein kinase 6	RLK
Macma4_03_g31330.1	Cysteine-rich receptor-like protein kinase 6	RLK
Macma4_03_g31510.1	Cysteine-rich receptor-like protein kinase 6	RLK
Macma4_03_g31480.1	Cysteine-rich receptor-like protein kinase 6	RLK
Macma4_03_g31540.1*	Cysteine-rich receptor-like protein kinase 6	RLK
Macma4_03_g25670.1	G-type lectin S-receptor-like serine/threonine kinase At1g11330	RLK
Macma4_03_g32260.1*	Leaf rust 10 resistance locus receptor-like kinase-like 1.2	RLK
Macma4_03_g32230.1*	Leaf rust 10 resistance rocus receptor-like kinase-like 1.2	RLK
Macma4_03_g32280.1	Leaf rust 10 resistance rocus receptor-like kinase-like 1.2	RLK
Macma4_03_g32270.1*	Leaf rust 10 resistance rocus receptor-like kinase-like 1.3	RLK
Macma4_03_g32220.1	Leaf rust 10 resistance rocus receptor-like kinase-like 2.1	RLK
Macma4_03_g32240.1	Leaf rust 10 resistance rocus receptor-like kinase-like 2.1	RLK
Macma4_03_g26300.1*	Leaf rust 10 resistance rocus receptor-like kinase-like 2.3	RLK
Macma4_03_g26540.1	LRR receptor-like serine/threonine-protein kinase ERL2	RLK
Macma4_03_g31350.1*	LRR receptor-like serine/threonine-protein kinase GSO1	RLK
Macma4_03_g31320.1*	LRR receptor-like serine/threonine-protein kinase GSO1	RLK
Macma4_03_g29270.1	Receptor-like serine/threonine-protein kinase At3g01300	RLK
Macma4_03_g24460.1	Receptor protein-tyrosine kinase CEPR2	RLK
Macma4_03_g25180.1	Receptor protein kinase TMK1	RLK
Macma4_03_g28830.1	Protein kinase domain-containing protein	RLK
Macma4_03_g29810.1*	Protein kinase domain-containing protein	RLK
Macma4_03_g30040.1	Protein kinase domain-containing protein	RLK
Macma4_03_g30400.1	Protein kinase domain-containing protein	RLK
Macma4_03_g30950.1	Protein kinase domain-containing protein	RLK

Macma4_03_g31060.1	Protein kinase domain-containing protein	RLK
Macma4_03_g31640.1*	Receptor-like protein EIX2	RLP
Macma4_03_g31660.1*	Receptor-like protein EIX2	RLP
Macma4_03_g31580.1	Receptor-like protein EIX2	RLP
Macma4_03_g31530.1	Receptor-like protein EIX2	RLP
Macma4_03_g31410.1	Receptor-like protein EIX2	RLP
Macma4_03_g31450.1	Receptor-like protein EIX2	RLP
Macma4_03_g31520.1*	Receptor-like protein EIX2	RLP
Macma4_03_g31610.1	Receptor-like protein EIX2	RLP
Macma4_03_g31460.1*	Receptor-like protein EIX2	RLP
Macma4_03_g31630.1*	Receptor-like protein EIX2	RLP
Macma4_03_g32400.1	Receptor-like protein EIX2	RLP
Macma4_03_g31550.1*	Receptor-like protein EIX2	RLP
Macma4_03_g31370.1	Receptor-like protein EIX2	RLP
Macma4_03_g31470.1	Receptor-like protein EIX2	RLP
Macma4_03_g31590.1	Receptor-like protein EIX2	RLP
Macma4_03_g31570.1*	Receptor-like protein EIX2	RLP
Macma4_03_g31500.1	Receptor-like protein EIX2	RLP
Macma4_03_g30450.1^	Disease resistance protein (CC-NBS-LRR)	NBS-LRR
Macma4_03_g30580.1*^	Disease resistance protein (CC-NBS-LRR)	NBS-LRR
Macma4_03_g30500.1*^	Disease resistance protein (CC-NBS-LRR)	NBS-LRR
Macma4_03_g30560.1*^	Disease resistance protein (CC-NBS-LRR)	NBS-LRR
Macma4_03_g30530.1*^	Disease resistance protein (CC-NBS-LRR)	NBS-LRR
Macma4_03_g30590.1^	Disease resistance protein (CC-NBS-LRR)	NBS-LRR
Macma4_03_g30490.1*^	Disease resistance protein (CC-NBS-LRR)	NBS-LRR
Macma4_03_g30510.1*^	Disease resistance protein (CC-NBS-LRR)	NBS-LRR
Macma4_03_g30840.1*^	Disease resistance protein (CC-NBS-LRR)	NBS-LRR
Macma4_03_g30470.1*^	NB-ARC domain-containing protein	NBS
Macma4_03_g30480.1^	NB-ARC domain-containing protein	NBS
Macma4_03_g24770.1*^	Disease resistance RPP13-like protein 1	NBS-LRR
Macma4_03_g24780.1^	Disease resistance RPP13-like protein 1	NBS-LRR
Macma4_03_g24800.1^	Disease resistance RPP13-like protein 1	NBS-LRR
Macma4_03_g24810.1*^	Disease resistance RPP13-like protein 1	NBS-LRR
Macma4_03_g24820.1*^	Disease resistance RPP13-like protein 1	NBS-LRR
Macma4_03_g24830.1*^	Disease resistance RPP13-like protein 1	NBS-LRR
Macma4_03_g24850.1*^	Disease resistance RPP13-like protein 1	NBS-LRR
Macma4_03_g24860.1^	Disease resistance RPP13-like protein 1	NBS-LRR
Macma4_03_g24880.1*^	Disease resistance RPP13-like protein 1	NBS-LRR
Macma4_03_g24890.1^	Disease resistance RPP13-like protein 1	NBS-LRR
Macma4_03_g24910.1^	Disease resistance RPP13-like protein 1	NBS-LRR
Macma4_03_g24930.1^	Disease resistance RPP13-like protein 1	NBS-LRR
Macma4_03_g30600.1^	Disease resistance RPP13-like protein 1	NBS-LRR
Macma4_03_g24870.1^	Disease resistance protein RGA1	NBS-LRR
Macma4_03_g24920.1*^	Disease resistance protein RGA1	NBS-LRR
Macma4_03_g30570.1^	Disease resistance protein RGA1	NBS-LRR
Macma4_03_g30440.1*^	Disease resistance protein RGA2	NBS-LRR
Macma4_03_g30460.1*^	Disease resistance protein RGA3	NBS-LRR
Macma4_03_g27700.1^	Disease resistance protein RGA4	NBS-LRR
Macma4_03_g30540.1^	NBS-type disease resistance protein	NBS
Macma4_03_g30550.1^	conserved hypothetical protein	NBS
Macma4_03_g24790.1^	Putative disease resistance protein At3g14460	AC
Macma4_03_g28590.1*	BTB/POZ domain and ankyrin repeat-containing protein NPR1	ANK
